# Evaluating the Canadian Packaged Food Supply Using Health Canada’s Proposed Nutrient Criteria for Restricting Food and Beverage Marketing to Children

**DOI:** 10.3390/ijerph17041250

**Published:** 2020-02-15

**Authors:** Christine Mulligan, Anthea K. Christoforou, Laura Vergeer, Jodi T. Bernstein, Mary R. L’Abbé

**Affiliations:** Department of Nutritional Sciences, Faculty of Medicine, University of Toronto, Toronto, ON M5S 1A8, Canada; christine.mulligan@mail.utoronto.ca (C.M.); anthea.christoforou@mail.utoronto.ca (A.K.C.); laura.vergeer@mail.utoronto.ca (L.V.); jodi.bernstein@mail.utoronto.ca (J.T.B.)

**Keywords:** marketing to children, food advertising, food marketing, marketing restrictions, nutrient profiling, nutrient composition, food policy, public health policy

## Abstract

Federally mandated restrictions on food and beverage marketing to kids (M2K) have been re-introduced as a national public health priority in Canada by the newly elected government, following the failure to implement a similar policy first proposed in 2016. This study examined the extent to which Canadian packaged foods, including products already displaying M2K on the packaging, would be permitted to be marketed, based on the nutrient criteria for marketing restrictions defined by Health Canada (in December 2018) as part of the previous policy proposal. Products from the University of Toronto Food Label Information Program 2013 database (n = 15,200) were evaluated using Health Canada’s published criteria: thresholds for sodium, sugars and saturated fats that products cannot exceed in order to be M2K. The proportion of products exceeding no thresholds (i.e., permitted to be M2K), the number of thresholds exceeded, and the proportion exceeding each individual threshold were calculated overall and in the subsample of products displaying M2K on the packaging (n = 747). Overall, 18.0% of products would be permitted to be M2K, versus 2.7% of products displaying M2K. Sodium was the most exceeded threshold overall (57.5% of products), whereas sugars was the most exceeded by products displaying M2K (80.1%). Only 4.7% of all products versus 10.4% of products displaying M2K exceeded all three thresholds. These results highlight the importance of reintroducing federal regulations restricting M2K in Canada and including marketing on product packaging in the regulatory scope.

## 1. Introduction

Marketing to kids (M2K) for foods and beverages higher in fat, sugars, and sodium (HFSS) is contributing to the poor dietary habits of children through its persuasive influence on their taste preferences, purchase requests and consumption patterns—ultimately intensifying the burden of childhood obesity [1,2]. It is estimated that globally, more than 150 million children are living with obesity, with this number expected to continue rising over the next decade [3]. In response to this public health problem, the World Health Organization (WHO) has made recommendations to limit the promotion of HFSS foods to children [4]. There have been varying degrees of implementation of these recommendations in different countries, with several countries having some form of voluntary, industry-led M2K restrictions (e.g., Australia, New Zealand, United States), and an increasing number of countries enforcing some version of mandatory M2K restrictions (e.g., Chile, United Kingdom, Sweden, Mexico, South Korea, Taiwan, Ireland) [5]. Furthermore, a number of countries have implemented mandatory front-of-pack labels and have prohibited foods bearing these labels from being M2K. In countries that have implemented mandatory federal restrictions, there is evidence suggesting a reduction in children’s exposure to marketing for HFSS foods. For example, in the United Kingdom, it is estimated that children saw 37% less HFSS television advertising following the implementation of restrictions and more recently, in Chile, almost 30% less HFSS breakfast cereals were found to display child-appealing marketing on their packaging post-implementation [6,7].

In Canada, there are mandatory M2K restrictions in the province of Québec, while in the rest of the country, the Canadian Children’s Food and Beverage Advertising Initiative (CAI), a voluntary, industry-led program is in place. However, both have been criticized for having limited effectiveness in restricting children’s exposure to HFSS foods due to loopholes such as limited or loosely defined criteria for the marketing media that are considered under the scope of the restrictions (e.g., lenient children’s viewership thresholds, or product packaging not being included), or in the case of the CAI, its voluntary nature [8,9,10,11]. 

In attempt to better protect Canadian children from the negative impacts of advertising, federally mandated restrictions on the marketing of HFSS foods to children under the age of 13 were proposed in 2016 as Bill S-228: the Child Health Protection Act, an amendment to the Canadian Food and Drugs Act [12]. The process of obtaining final parliamentary approval of the Bill was stalled prior to the 2019 Canadian federal election, and the Bill effectively died. Following his re-election, the Prime Minister has recently published his mandate letter to the Minister of Health, setting the implementation of new restrictions on the commercial marketing of foods and beverages to children as a key public health priority for Canada over the next four years [13]. 

Prior to the failure of Bill S-228, Health Canada released a draft “Guide to the Application of the Child-Health Protection Act (Bill S-228)” (henceforth referred to as the “Guide”) in December 2018 [14]. The Guide outlined the proposed regulatory approach for the implementation of the M2K restrictions, including a description of the types of advertising that would be considered “primarily directed at children” (e.g., through the setting or design of the advertising) and the types of foods that are appropriate to be advertised to children, based on the product’s nutritional composition [14]. For the latter, Health Canada proposed a set of nutrient criteria (i.e., a nutrient profile (NP) model), using thresholds for sugars, sodium and saturated fats that would determine which products would be permitted or restricted from being M2K [14]. While some studies to date have tested earlier versions of these nutrient criteria in limited samples of Canadian products displaying M2K on the packaging [15,16], the broad impact of the application of these thresholds on the entire Canadian food supply has yet to be investigated using the most up-to-date publicly available proposed nutrient criteria (i.e., those published in the draft Guide). Moreover, a thorough understanding of the potential impacts of the previously proposed restrictions could provide the empirical evidence needed to facilitate and expedite the development and implementation of the new M2K restrictions. Importantly as well, despite evidence that product packaging is children’s top source of exposure to food marketing [17], M2K on product packaging was not included under the scope of the proposed regulations described in the Guide, and would not be restricted. Evidence to support the expansion of the regulatory scope to include this important marketing medium is warranted. 

The objectives of this study were therefore to evaluate Canadian packaged food products, including those that currently display M2K on the packaging, in terms of the product’s eligibility to be M2K, according to Health Canada’s previously proposed nutrient criteria for advertising restrictions, if the legislation were to include marketing on product packaging. 

## 2. Materials and Methods 

Anlyses were conducted using the University of Toronto Food Label Information Program (FLIP) 2013 database, described in detail elsewhere [18]. Briefly, FLIP 2013 is a branded food composition database containing nutritional information for 15,342 unique food products from the four largest national grocery retailers in Canada (Loblaws, Metro, Sobeys, and Safeway), representing approximately 75% of the Canadian retail food market share at the time of collection. FLIP contains information such as a product’s Nutrition Facts table (NFt), ingredients list, price, company and brand name, as well as photos of all sides of product packages. Nutritional information was recorded for products in the “as sold” form and calculated for the “as consumed” form, when necessary (e.g., condensed soup). Foods in FLIP 2013 were classified into 24 major and 153 minor categories as defined in the Table of Reference Amounts for Foods (TRA) in the Food and Drug Regulations, which provides detailed examples of the types of foods in each category and subcategory [19]. The identification of products displaying M2K on the packaging has been previously determined in FLIP 2013, based on the display of at least one of the following persuasive marketing techniques: children’s product lines (e.g., junior, mini); child-appealing lettering, images or graphics; allusions to fun or play; unconventional flavors, colors, or shapes; toys, coupons, prizes, or contests; games; and child-appealing characters [20]. 

In order to evaluate products’ eligibility to be M2K, this study used the latest publicly available nutrient criteria released by Health Canada in December 2018 in the draft Guide [14]. These criteria require the assessment of products on a nutrient-by-nutrient basis by using existing “low in” nutrient content claim (NCC) thresholds [21] for products containing “sugars”, “added sodium” or “added fats”. In other words, only products that contained “sugars” were evaluated according to the “low in sugars” NCC threshold, only products that contained “added sodium” were evaluated according to the “low in sodium” NCC threshold, and only products that contained “added fat” were evaluated according to the “low in saturated fat” NCC threshold.

The draft Guide defines “sugars” as: “sugars, except those naturally present in fruits or vegetables—whole or cut—that are fresh, frozen, canned or dried; dairy products; grains; legumes; or nuts and seeds” [14]. This definition is consistent with the WHO definition of free sugars [22], the presence of which has been previously determined for products in FLIP 2013, and was used in the current analyses [18]. Therefore, only products containing any free sugars ingredient were evaluated using the “low in sugars” NCC threshold and those without free sugars ingredients were considered “exempt” from evaluation. 

“Added sodium” is defined as: “salt, other sodium salts or ingredients that contain sodium that functionally substitute for added salt”, when added to a product [14]. This definition is consistent with the criteria for a product to carry a “no added sodium” or “no added salt” NCC [21]. Based on this definition, product ingredient lists were analyzed for the presence of “added sodium” and products containing “added sodium” were evaluated using the “low in sodium” NCC threshold and those without added sodium were considered “exempt” from evaluation. If the product was a stand-alone salt product (e.g., celery salt, table salt, garlic salt) and contained no other added sources of sodium, this product was not considered to contain “added sodium”, as per the criteria outlined in the Guide. However, where these products were added to other products, they were considered an “added sodium” ingredient (e.g., garlic salt added to chicken nuggets). 

Lastly, the draft Guide defines “added fat” as: “fats or oils set out in Division 9 of the Food and Drug Regulations; butter; ghee; or ingredients that contain added fats or oils, butter, or ghee” when added to a product. This definition is consistent with the “no added fat” NCC [21]. Based on this definition, product ingredient lists were analyzed for the presence of “added fat” and products containing “added fat” ingredients were evaluated under the “low in saturated fat” NCC threshold and those without “added fat” were considered “exempt” from evaluation. If the product was a stand-alone fat or oil product (e.g., olive oil, butter) it was not considered to be a source of “added” fat. 

Table 1 shows the “low in” NCC thresholds under which each product was evaluated. For products requiring preparation (e.g., pudding mix), the thresholds were applied to the “as consumed” nutrient information, otherwise, “as sold” nutrient values were used. The nutrient content of a product was evaluated using the largest of either the TRA reference amount or the manufacturer stated serving size. Additionally, the draft Guide outlines different nutrient thresholds for foods vs. main dishes due to the larger reference amounts of main dish product; thus, “Combination dishes” in TRA subcategories N.1 and N.2 [19] were classified as “main dishes” and all other products were classified as “foods”. Products exceeding any of the respective thresholds under which they were evaluated would be restricted from M2K. Products which were exempt from all thresholds or did not exceed any thresholds would be permitted to be M2K. 

A total of 141 products were excluded from the analyses because they were products indicated for special dietary use (i.e., TRA category X—Meal replacements, n = 55), because of errors in nutrient declarations in the NFt, as determined by Atwater calculations that varied >20% from declared caloric values (n = 55), or because products had missing nutrient information for one or more of the three relevant nutrients (n = 31). The final analysis included 15,200 products from 22 food categories, and the sub-analysis of products currently displaying M2K on the packaging included 747 products in 16 food categories, representing 4.9% of the total analytic sample. 

The number and proportion of products that would be permitted and restricted from M2K were calculated. The number and proportion of products exceeding the “low in” threshold for each individual nutrient as well as the total number of thresholds a product exceeded were also calculated. Analyses were completed for the entire sample, as well as for the subsample of products displaying M2K on the package, for all major TRA food categories.

## 3. Results

Overall, 13.4% of packaged food products would be exempt from all “low in” thresholds (i.e., did not contain any “sugars”, “added sodium” or “added fats”), and 4.5% of products did not exceed any “low in” threshold, for a total 18.0% of products that would be permitted to be M2K (Table 2). In the sub-sample of products displaying M2K on the packaging, 0.9% of products would be exempt from all nutrient thresholds and 1.7% did not exceed any of the thresholds. Thus, 97.3% (n = 727) of foods displaying M2K on the packaging would be restricted from M2K.

Out of all products, very few food categories (4/22 categories) had greater than 50% of products that would be permitted to be M2K: eggs and egg substitutes (83.9%), nuts and seeds (82.4%), legumes (71.7%) and cereals and grain products (64.3%). Not surprisingly, these categories also had high proportions of products that would be exempt from evaluation under the “low in” thresholds due to the absence of added sugars, sodium or fats. However, almost half of all food categories (10/22 categories) had greater than 90% of products that would be restricted from M2K, including: packaged salads (100%); dessert toppings and fillings (99.0%); sugars and sweets (98.6%); meat, poultry, their products and substitutes (98.2%); combination dishes (97.6%); bakery products (96.2%); soups (95.8%); desserts (95.6%); sauces, dips, gravies and condiments (94.0%); and snacks (91.1%). 

For products currently displaying M2K on the packaging, >90% of products in all food categories would be restricted from M2K, and in most (9/16) food categories, 100% of products would be restricted. 

When examining the number of individual thresholds that were exceeded, in the overall sample, 18.0% of products did not exceed any “low in” threshold, 53.3% of products exceeded one threshold, 24.1% exceeded two thresholds, and 4.7% exceeded all three thresholds (Table 3). Categories that had the most products exceeding all three nutrient thresholds were bakery products (21.5%) and packaged salads (14.3%). For products displaying M2K on the packaging, 2.7% of products did not exceed any “low in” threshold, 53.1% of products exceeded 1 threshold, 33.7% exceeded 2, and 10.4% exceeded all three thresholds. In this sub-sample, bakery products, combination dishes, and beverages had the most products exceeding all three thresholds (27.2%, 21.9% and 18.2%, respectively). 

Overall, across all food categories, 38.9% exceeded the sugars threshold, 57.5% of products exceeded the sodium threshold, and 19.1% exceeded the saturated fat threshold (Table 4). Saturated fats also had the highest proportion of products exempt from evaluation (50.2%). Not surprisingly, the categories with the highest proportion of products that would exceed “low in” thresholds varied by nutrient. 

Of foods currently displaying M2K on the packaging, 43.6% exceeded the sodium threshold, 80.1% of products exceeded the sugars threshold, and 28.2% exceeded the saturated fat threshold. Like the main analysis, saturated fat also had the highest proportion of products that would be exempt from evaluation under that threshold (39.6%), and the categories most exceeding each threshold varied by nutrient. 

## 4. Discussion

The results of this study indicate that Health Canada’s proposed nutrient criteria would restrict M2K for most packaged food products and restrict the marketing of virtually all products that currently display M2K on the package—if the regulations were to consider product packaging under the scope of the included marketing platforms. Moreover, compared to products overall, products displaying M2K on the packaging, had an almost 7-fold lower proportion (2.7% vs. 18.0%) of products not exceeding any nutrient threshold and therefore being permitted to be M2K. Similarly, products displaying M2K on the packaging had a much higher proportion of products exceeding two nutrient thresholds and the proportion that exceeded all three nutrient thresholds was more than double that of the overall sample (i.e., 10.4% vs. 4.7%). These results align with other research noting the elevated levels of fat, sugars and sodium in food products that are M2K in Canada and supports the need for the implementation of regulations restricting M2K to limit the harmful impacts of this marketing practice [8,20,23].

When looking at nutrient thresholds individually, different results can be seen between the overall sample and the subsample of products being M2K. Overall, sodium was the added nutrient that most frequently exceeded the “low in” threshold, but in products displaying M2K, sugars were the most exceeded threshold. This is consistent with the literature showing that products displaying M2K are more likely to contain excessive levels of total and free sugars [24,25]. Importantly, the food categories that have the greatest proportions of products exceeding the sugars and saturated fat thresholds are categories that make up a large proportion of the total sample of products displaying M2K (e.g., bakery products, desserts, sugars and sweets, and beverages). These are also among categories that account for over one-third of children’s total sugars intakes in Canada, and are large contributors to children’s overall caloric intake [26,27]. Moreover, bakery (e.g., cookies, cakes, grain-based bars) and beverage products (e.g., carbonated and non-carbonated drinks) were also in the top categories exceeding all three nutrient thresholds, suggesting that these categories are an area of particular concern. Conversely, categories with a high proportion of products displaying M2K exceeding the sodium threshold represent a relatively small overall proportion of this subsample (e.g., marine and freshwater animals; meat, poultry, their products and substitutes; potatoes, sweet potatoes and yams; soups). These results suggest that in categories where M2K is particularly pervasive, while high sodium contents should not be ignored, sugars and saturated fats may be the primary nutrients of concern for Canadian children and could be key targets for product reformulation.

This analysis also allows for the comparison of the proposed Health Canada nutrient thresholds to other NP models developed, specifically for the purpose of restricting M2K, which have been applied to the same sample (i.e., FLIP 2013). The Health Canada nutrient criteria are less stringent than the Pan American Health Organization (PAHO) NP model (permitting 15.8% of products), but more stringent than the WHO Regional Office for Europe (EURO) model (29.8% permitted), the voluntary, industry-led CAI Uniform Nutrition Criteria (25.3%) and the Food Standards Australia New Zealand Nutrient Profiling Scoring Criterion (FSANZ-NPSC) (49.0%) [8,20]. In the subsample of products displaying M2K on the packaging, the Health Canada criteria was the most stringent of all models, with the PAHO model permitting 3.5% of products, EURO permitting 6.2%, the CAI permitting 23.4% and FSANZ-NPSC permitting 24.4% [8,20]. However, all models would restrict a higher proportion of products displaying M2K on the packaging than the overall sample, confirming that no matter the nutrient criteria used to assess a product’s eligibility for M2K, these products tend to be less healthful than the overall food supply.

Key characteristics of the Health Canada NP model can help explain its stringency relative to other models, specifically it’s consideration of only ‘negative’ nutrients (e.g., sodium, sugars, saturated fat) and that it does not use category-specific criteria, requiring all foods to meet the same nutrient thresholds. Health Canada’s model performs similarly to the PAHO model, which also only applies ‘negative’ nutrient thresholds to processed and ultra-processed foods (similarly to having “added ingredients”) [28]. Conversely, the EURO and CAI models both employ category-specific nutrient criteria which take into account the nature of a product category and adjust the nutrient thresholds accordingly (e.g., higher fat thresholds for dairy products), which could explain these models’ reduced stringency compared to Health Canada’s thresholds [29,30]. The FSANZ-NPSC scores products based on both ‘negative’ and ‘positive’ nutrients (e.g., protein, fiber), whereby the addition of positive nutrients can balance high levels of negative nutrients and increase a product’s final score [31]. While this allows for the consideration of health-promoting aspects of a product, it reduces this model’s stringency in terms of reducing children’s exposure to nutrients of public health concern, compared to Health Canada’s criteria. It is worth noting that although the FSANZ-NPSC was based on the Ofcom model which was developed for restricting M2K in the UK [32], its primary purpose was not for M2K restrictions, rather for determining a product’s eligibility to carry health claims [31].

While the proposed nutrient criteria would be effective in ensuring that only products with low levels of sugars, sodium and saturated fats are permitted to be M2K, there are some key limitations to the regulatory framework outlined in the draft Guide that may impact the overall strictness of the policy. The draft Guide explains that a product must first be determined to be “directed primarily at children” before being subject to the proposed nutrient criteria. However, the criteria that Health Canada has proposed to determine M2K are highly subjective, leaving several loopholes for marketing manipulation and therefore, the evasion of the highly stringent nutrient criteria. As mentioned, a key gap in these criteria is the failure to include product packaging under the scope of child-appealing marketing media, despite it being the largest source of children’s exposure to food marketing [17]. The present analysis demonstrates that 97.3% of products that are currently being M2K on product packaging would not meet the proposed nutrient criteria, but if the regulations were to be implemented as is, all of these products would still be permitted to be M2K, despite containing elevated levels of nutrients of public health concern. In order to ensure that future M2K restrictions are effective in reducing children’s exposure to HFSS foods, they should either require that all foods meet the proposed nutrient criteria before being eligible to be M2K, or broaden and clarify the definition of marketing “directed primarily at children”. However, the presented study finds that overall, the Health Canada’s proposed criteria would restrict 1.4 times more products from M2K than the CAI’s Uniform Nutrition Criteria and would restrict 8.6 times more products from M2K in the subsample currently displaying M2K on the packaging [8]. This corroborates previous criticisms of the CAI’s ability to effectively restrict M2K and demonstrates that the nutrient criteria proposed by Health Canada would be a vast improvement over the voluntary industry-led M2K restrictions that currently exist in Canada, particularly if the previously discussed limitations of the proposed regulations are addressed in the future iteration.

This study presents the first analysis of the most recently published version of Health Canada’s proposed nutrient criteria to support the marketing restrictions outlined in Bill S-228, using a large, nationally representative sample of Canadian packaged products. This work also facilitates the comparison of Health Canada’s proposed nutrient criteria to several different NP models and can assist policy makers and researchers in elucidating the strengths and limitations of various NP models for restricting children’s exposure to HFSS foods. There are some limitations to this analysis inherent to the nature FLIP database, namely that it does not contain information for fresh and unpackaged food products (e.g., fresh fruits and vegetables) which would largely be permitted to be M2K. Moreover, FLIP is a cross-sectional database and therefore, only represents the status of the Canadian packaged food supply at the time of collection. Additionally, FLIP 2013 data is not sales-weighted and analyses on Canadians’ purchasing (and consequently, consumption) of packaged products were not within the scope of this research. Finally, this study only presents data on products that display M2K on the packaging and therefore, that subsample of products likely underestimates the proportion of products across the entire food supply that are advertised to children on all possible marketing platforms, such as television or the internet.

## 5. Conclusions

Overall, the results of this work suggest that Health Canada’s previously proposed nutrient criteria are stringent and have strong potential to reduce children’s exposure to marketing of HFSS foods and beverages. The results also demonstrate that products displaying M2K on the packaging are more likely to exceed thresholds for nutrients of public health concern than the overall food supply and regulations are needed to ensure that all forms of child-appealing marketing are covered under the scope of regulations in order to best protect Canadian children, and children in other countries aiming to regulate similarly. Ultimately, this research supports the necessity of continued efforts to implement federally mandated restrictions on M2K in Canada and globally to help improve children’s diets and reduce their risk of diet-related chronic disease.

## Figures and Tables

**Table 1:** Summary of proposed “low in” thresholds for sugars, sodium and saturated fats ^a,b^.

Foods (Other than Main Dishes with a Reference Amount (RA) above 200 g)	Main Dishes (with an RA above 200 g)
≤5 g total sugar per RA or Serving Size (SS), whichever is the greater OR per 50 g of the product, if the RA of the product is 30 g or 30 mL or less	≤5 g total sugar per 100 g
≤140 mg sodium per RA or SS, whichever is the greater OR per 50 g of the product, if the RA of the product is 30 g or 30 mL or less	≤140 mg sodium per 100 g
≤2 g saturated fatty acid (SFA) + trans fatty acid (TFA) per RA or SS, whichever is the greater and ≤15% energy from the sum of SFA+TFA	≤2 g SFA + TFA per 100 g and ≤ 15% energy from the sum of SFA+TFA

^a^ Proposed thresholds for sugars, sodium and saturated fats levels are based on existing Food and Drug Regulations thresholds for “low in” nutrient content claims [21]. ^b^ If a product had added sugars, sodium or fats identified in the ingredients list, it was subject to evaluation under the “low in” threshold for the identified “added” nutrient. If a product exceeded any threshold, it would be restricted from M2K. Products with no added sugars, sodium or fats were exempted from the threshold for that nutrient. When it was impossible to determine if the product was below the threshold for all relevant nutrients (i.e., due to missing nutrient information), the product was excluded from the analysis.

**Table 2:** Number (n) and proportion (%) of products that would be permitted or restricted from marketing to children (M2K).

Food Category ^a^	Analytic Sample ^b^	Total Products Analyzed		Permitted to Be M2K						Restricted from M2K	
				Exempt from Evaluation ^c^		Not Exempt from Evaluation and Does Not Exceed “Low in” Thresholds		Total Products Permitted for M2K		Not Exempt from Evaluation and Exceeds “Low in” Thresholds	
		n	% ^d^	n	% ^e^	n	% ^e^	n	% ^e^	n	% ^e^
**Total**	All	15200	100.0	2039	13.4	691	4.5	2730	18.0	12470	82.0
	M2K	747	100.0	7	0.9	13	1.7	20	2.7	727	97.3
Bakery Products (e.g., bread, cookies, grain-based bars)	All	2085	13.7	9	0.4	70	3.4	79	3.8	2006	96.2
	M2K	173	23.2	0	0.0	6	3.5	6	3.5	167	96.5
Beverages (e.g., carbonated and non-carbonated drinks)	All	482	3.2	122	25.3	77	16.0	199	41.3	283	58.7
	M2K	11	1.5	0	0.0	1	9.1	1	9.1	10	90.9
Cereals and other grain products (e.g., breakfast cereals, pasta)	All	1028	6.8	638	62.1	23	2.2	661	64.3	367	35.7
	M2K	51	6.8	1	2.0	0	0.0	1	2.0	50	98.0
Dairy products and substitutes (e.g., milk, yogurt)	All	1221	8.0	135	11.1	115	9.4	250	20.5	971	79.5
	M2K	74	9.9	0	0.0	0	0.0	0	0.0	74	100.0
Desserts (e.g., ice cream, puddings)	All	827	5.4	5	0.6	31	3.7	36	4.4	791	95.6
	M2K	144	19.3	0	0.0	5	3.5	5	3.5	139	96.5
Dessert toppings and fillings (e.g., cake frosting)	All	104	0.7	0	0.0	1	1.0	1	1.0	103	99.0
	M2K	7	0.9	0	0.0	0	0.0	0	0.0	7	100.0
Eggs and egg substitutes (e.g., omelet mix)	All	56	0.4	47	83.9	0	0.0	47	83.9	9	16.1
	M2K	0	0.0								
Fats and oils (e.g., dressings, mayonnaise)	All	535	3.5	168	31.4	11	2.1	179	33.5	356	66.5
	M2K	0	0.0								
Marine and freshwater animals (e.g., fish sticks, shrimp)	All	440	2.9	56	12.7	35	8.0	91	20.7	349	79.3
	M2K	2	0.3	0	0.0	0	0.0	0	0.0	2	100.0
Fruit and fruit juices (e.g., applesauce, canned fruit)	All	1088	7.2	175	16.1	48	4.4	223	20.5	865	79.5
	M2K	58	7.8	5	8.6	0	0.0	5	8.6	53	91.4
Legumes (e.g., beans, tofu)	All	180	1.2	104	57.8	25	13.9	129	71.7	51	28.3
	M2K	0	0.0								
Meat, poultry, their products and substitutes (e.g., chicken nuggets, sandwich meats)	All	899	5.9	15	1.7	1	0.1	16	1.8	883	98.2
	M2K	9	1.2	0	0.0	0	0.0	0	0.0	9	100.0
Miscellaneous category (e.g., spices, culinary ingredients)	All	473	3.1	64	13.5	37	7.8	101	21.4	372	78.6
	M2K	14	1.9	0	0.0	0	0.0	0	0.0	14	100.0
Combination fishes (e.g., frozen burritos, pizza)	All	1304	8.6	0	0.0	31	2.4	31	2.4	1273	97.6
	M2K	64	8.6	0	0.0	0	0.0	0	0.0	64	100.0
Nuts and seeds (e.g., peanut butter)	All	210	1.4	160	76.2	13	6.2	173	82.4	37	17.6
	M2K	10	1.3	0	0.0	0	0.0	0	0.0	10	100.0
Potatoes, sweet potatoes and yams (e.g., French fries)	All	140	0.9	4	2.9	21	15.0	25	17.9	115	82.1
	M2K	4	0.5	0	0.0	0	0.0	0	0.0	4	100.0
Salads (e.g., Greek or macaroni)	All	70	0.5	0	0.0	0	0.0	0	0.0	70	100.0
	M2K	0	0.0								
Sauces, dips, gravies and condiments (e.g., ketchup, hummus)	All	1224	8.1	56	4.6	18	1.5	74	6.0	1150	94.0
	M2K	0	0.0								
Snacks (e.g., popcorn, chips)	All	813	5.3	15	1.8	57	7.0	72	8.9	741	91.1
	M2K	41	5.5	0	0.0	1	2.4	1	2.4	40	97.6
Soups (i.e., all varieties)	All	454	3.0	1	0.2	18	4.0	19	4.2	435	95.8
	M2K	1	0.1	0	0.0	0	0.0	0	0.0	1	100.0
Sugars and sweets (e.g., confectionary, chocolate, syrup)	All	734	4.8	5	0.7	5	0.7	10	1.4	724	98.6
	M2K	84	11.2	1	1.2	0	0.0	1	1.2	83	98.8
Vegetables (e.g., canned or frozen)	All	833	5.5	260	31.2	54	6.5	314	37.7	519	62.3
	M2K	0	0.0								

^a^ Foods in FLIP 2013 were classified into food categories as defined in the Table of Reference Amounts for Foods (TRA) in the Food and Drug Regulations [19]; ^b^ All = all products in FLIP (n = 15,200); M2K = products displaying child-appealing marketing on the packaging (n = 747); ^c^ Products that contained no “sugars”, “added sodium” or “added fat” were exempted from all “low in” thresholds; ^d^ Percentage of total products analyzed in the analytic sample (i.e., out of n = 15,200 for “All” and out of n = 747 for “M2K”), note: some rows may sum to 100% plus or minus 0.1%, due to rounding; ^e^ Percentage of total products analyzed in that food category, in that analytic sample.

**Table 3:** Number (n) and proportion (%) of products exceeding zero, one, two or three “low in” thresholds.

Food Category ^b^	Analytic Sample ^c^	Total Products Analyzed		Number of “Low in” Thresholds Exceeded ^a^							
				0		1		2		3	
		n	% ^d^	n	% ^e^	n	% ^e^	n	% ^e^	n	% ^e^
**Total**	All	15200	100.0	2730	18.0	8097	53.3	3662	24.1	711	4.7
	M2K	747	100.0	20	2.7	397	53.1	252	33.7	78	10.4
Bakery Products (e.g., bread, cookies, grain-based bars)	All	2085	13.7	79	3.8	956	45.9	602	28.9	448	21.5
	M2K	173	23.2	6	3.5	51	29.5	69	39.9	47	27.2
Beverages (e.g., carbonated and non-carbonated drinks)	All	482	3.2	199	41.3	222	46.1	49	10.2	12	2.5
	M2K	11	1.5	1	9.1	7	63.6	1	9.1	2	18.2
Cereals and other grain products (e.g., breakfast cereals, pasta)	All	1028	6.8	661	64.3	149	14.5	210	20.4	8	0.8
	M2K	51	6.8	1	2.0	14	27.5	36	70.6	0	0.0
Dairy products and substitutes (e.g., milk, yogurt)	All	1221	8.0	250	20.5	857	70.2	104	8.5	10	0.8
	M2K	74	9.9	0	0.0	60	81.1	14	18.9	0	0.0
Desserts (e.g., ice cream, puddings)	All	827	5.4	36	4.4	400	48.4	344	41.6	47	5.7
	M2K	144	19.3	5	3.5	63	43.8	65	45.1	11	7.6
Dessert toppings and fillings (e.g., cake frosting)	All	104	0.7	1	1.0	73	70.2	30	28.8	0	0.0
	M2K	7	0.9	0	0.0	6	85.7	1	14.3	0	0.0
Eggs and egg substitutes (e.g., omelet mix)	All	56	0.4	47	83.9	9	16.1	0	0.0	0	0.0
	M2K	0	0.0								
Fats and oils (e.g., dressings, mayonnaise)	All	535	3.5	179	33.5	225	42.1	129	24.1	2	0.4
	M2K	0	0.0								
Marine and freshwater animals (e.g., fish sticks, shrimp)	All	440	2.9	91	20.7	287	65.2	56	12.7	6	1.4
	M2K	2	0.3	0	0.0	2	100.0	0	0.0	0	0.0
Fruit and fruit juices (e.g., applesauce, canned fruit)	All	1088	7.2	223	20.5	848	77.9	17	1.6	0	0.0
	M2K	58	7.8	5	8.6	53	91.4	0	0.0	0	0.0
Legumes (e.g., beans, tofu)	All	180	1.2	129	71.7	50	27.8	1	0.6	0	0.0
	M2K	0	0.0								
Meat, poultry, their products and substitutes (e.g., chicken nuggets, sandwich meats)	All	899	5.9	16	1.8	710	79.0	142	15.8	31	3.4
	M2K	9	1.2	0	0.0	6	66.7	3	33.3	0	0.0
Miscellaneous category (e.g., spices, culinary ingredients)	All	473	3.1	101	21.4	201	42.5	159	33.6	12	2.5
	M2K	14	1.9	0	0.0	6	42.9	7	50.0	1	7.1
Combination Dishes (e.g., frozen burritos, pizza)	All	1304	8.6	31	2.4	672	51.5	547	41.9	54	4.1
	M2K	64	8.6	0	0.0	23	35.9	27	42.2	14	21.9
Nuts and seeds (e.g., peanut butter)	All	210	1.4	173	82.4	21	10.0	16	7.6	0	0.0
	M2K	10	1.3	0	0.0	5	50.0	5	50.0	0	0.0
Potatoes, sweet potatoes and yams (e.g., French fries)	All	140	0.9	25	17.9	89	63.6	26	18.6	0	0.0
	M2K	4	0.5	0	0.0	3	75.0	1	25.0	0	0.0
Salads (e.g., Greek or macaroni)	All	70	0.5	0	0.0	36	51.4	24	34.3	10	14.3
	M2K	0	0.0								
Sauces, dips, gravies and condiments (e.g., ketchup, hummus)	All	1224	8.1	74	6.0	624	51.0	502	41.0	24	2.0
	M2K	0	0.0								
Snacks (e.g., popcorn, chips)	All	813	5.3	72	8.9	500	61.5	208	25.6	33	4.1
	M2K	41	5.5	1	2.4	30	73.2	8	19.5	2	4.9
Soups (i.e., all varieties)	All	454	3.0	19	4.2	278	61.2	150	33.0	7	1.5
	M2K	1	0.1	0	0.0	1	100.0	0	0.0	0	0.0
Sugars and sweets (e.g., confectionary, chocolate, syrup)	All	734	4.8	10	1.4	461	62.8	256	34.9	7	1.0
	M2K	84	11.2	1	1.2	67	79.8	15	17.9	1	1.2
Vegetables (e.g., canned or frozen)	All	833	5.5	314	37.7	429	51.5	90	10.8	0	0.0
	M2K	0	0.0								

^a^ Total number of nutrient thresholds a single products exceeded for sugars, sodium and saturated fats; ^b^ Foods in FLIP 2013 were classified into food categories as defined in the Table of Reference Amounts for Foods (TRA) in the Food and Drug Regulations [19]; ^c^ All = all products in FLIP (n = 15,200); M2K = products displaying child-appealing marketing on the packaging (n = 747); ^d^ Percentage of total products analyzed in the analytic sample (i.e., out of n = 15200 for “All” and out of n = 747 for “M2K”), note: some rows may sum to 100% plus or minus 0.1%, due to rounding; ^e^ Percentage of total products analyzed in that food category, in that analytic sample.

**Table 4:** Number (n) and proportion (%) of products exceeding each nutrient threshold.

Food Category ^b^	Analytic Sample ^c^	Total Products Analyzed		Sugars ^a^						Sodium ^a^						Saturated Fat ^a^					
				Exempt from Evaluation ^d^		Not Exempt, Does Not Exceed “Low in” Threshold		Not Exempt, Exceeds “Low in” Threshold		Exempt from Evaluation ^d^		Not Exempt, Does Not Exceed “Low in” Threshold		Not Exempt, Exceeds “Low in” Threshold		Exempt from Evaluation ^d^		Not Ees not Exceed “Low in” Threshold		Not Exempt, Exceeds “Low in” Threshold	
		n	% ^e^	n	% ^f^	n	% ^f^	n	% ^f^	n	% ^f^	n	% ^f^	n	% ^f^	n	% ^f^	n	% ^f^	n	% ^f^
**Total**	All	15200	100.0	5286	34.8	3995	26.3	5911	38.9	4264	28.1	2195	14.4	8736	57.5	7623	50.2	4660	30.7	2907	19.1
	M2K	747	100.0	61	8.2	88	11.8	598	80.1	194	26.0	227	30.4	326	43.6	296	39.6	240	32.1	211	28.2
Bakery Products (e.g., cookies, grain-based bars)	All	2085	13.7	289	13.9	625	30.0	1171	56.2	39	1.9	464	22.3	1582	75.9	155	7.4	1178	56.5	751	36.0
	M2K	173	23.2	9	5.2	22	12.7	142	82.1	1	0.6	52	30.1	120	69.4	0	0.0	105	60.7	68	39.3
Beverages (e.g., carbonated and non-carbonated drinks)	All	482	3.2	185	38.4	24	5.0	273	56.6	273	56.6	153	31.7	56	11.6	435	90.2	20	4.1	27	5.6
	M2K	11	1.5	1	9.1	0	0.0	10	90.9	1	9.1	8	72.7	2	18.2	8	72.7	0	0.0	3	27.3
Cereals and other grain products (e.g., breakfast cereals, pasta)	All	1028	6.8	715	69.6	46	4.5	267	26.0	679	66.1	91	8.9	258	25.1	802	78.0	157	15.3	68	6.6
	M2K	51	6.8	1	2.0	4	7.8	46	90.2	7	13.7	4	7.8	40	78.4	25	49.0	26	51.0	0	0.0
Dairy products and substitutes (e.g., milk, yogurt)	All	1221	8.0	763	62.5	84	6.9	374	30.6	321	26.3	262	21.5	638	52.3	1064	87.1	74	6.1	83	6.8
	M2K	74	9.9	12	16.2	4	5.4	58	78.4	37	50.0	8	10.8	29	39.2	73	98.6	0	0.0	1	1.4
Desserts (e.g., ice cream, puddings)	All	827	5.4	49	5.9	5	0.6	766	92.6	311	37.6	375	45.3	136	16.4	410	49.6	90	10.9	327	39.5
	M2K	144	19.3	3	2.1	4	2.8	137	95.1	47	32.6	76	52.8	21	14.6	64	44.4	12	8.3	68	47.2
Dessert toppings and fillings (e.g., cake frosting)	All	104	0.7	1	1.0	1	1.0	102	98.1	33	31.7	65	62.5	6	5.8	42	40.4	37	35.6	25	24.0
	M2K	7	0.9	0	0.0	0	0.0	7	100.0	5	71.4	2	28.6	0	0.0	5	71.4	1	14.3	1	14.3
Eggs and egg substitutes (e.g., omelet mix)	All	56	0.4	51	91.1	3	5.4	2	3.6	49	87.5	0	0.0	7	12.5	55	98.2	1	1.8	0	0.0
	M2K	0	0.0																		
Fats and oils (e.g., dressings, mayonnaise)	All	535	3.5	269	50.3	170	31.8	96	17.9	180	33.6	9	1.7	346	64.7	286	53.5	202	37.8	47	8.8
	M2K	0	0.0																		
Marine and fresh water animals (e.g., fish sticks, shrimp)	All	440	2.9	249	56.6	167	38.0	24	5.5	68	15.5	25	5.7	347	78.9	246	55.9	148	33.6	46	10.5
	M2K	2	0.3	1	50.0	1	50.0	0	0.0	0	0.0	0	0.0	2	100.0	0	0.0	2	100.0	0	0.0
Fruit and fruit juices (e.g., applesauce, canned fruit)	All	1088	7.2	220	20.2	9	0.8	859	79.0	974	89.5	96	8.8	18	1.7	1001	92.0	82	7.5	5	0.5
	M2K	58	7.8	5	8.6	0	0.0	53	91.4	40	69.0	18	31.0	0	0.0	58	100.0	0	0.0	0	0.0
Legumes (e.g., beans, tofu)	All	180	1.2	174	96.7	2	1.1	4	2.2	108	60.0	24	13.3	48	26.7	178	98.9	2	1.1	0	0.0
	M2K	0	0.0																		
Meat, poultry, their products and substitutes (e.g., chicken nuggets, sandwich meats)	All	899	5.9	201	22.4	614	68.3	84	9.3	15	1.7	1	0.1	883	98.2	605	67.3	174	19.4	120	13.3
	M2K	9	1.2	3	33.3	6	66.7	0	0.0	0	0.0	0	0.0	9	100.0	0	0.0	6	66.7	3	33.3
Miscellaneous category (e.g., spices, culinary ingredients)	All	473	3.1	151	31.9	129	27.3	193	40.8	90	19.0	44	9.3	339	71.7	214	45.2	236	49.9	23	4.9
	M2K	14	1.9	2	14.3	0	0.0	12	85.7	1	7.1	4	28.6	9	64.3	0	0.0	12	85.7	2	14.3
Combination Dishes (e.g., frozen burritos, pizza)	All	1304	8.6	216	16.6	946	72.5	141	10.8	5	0.4	35	2.7	1264	96.9	111	8.5	662	50.8	523	40.1
	M2K	64	8.6	9	14.1	38	59.4	17	26.6	0	0.0	0	0.0	64	100.0	5	7.8	21	32.8	38	59.4
Nuts and seeds (e.g., peanut butter)	All	210	1.4	167	79.5	20	9.5	23	11.0	171	81.4	10	4.8	29	13.8	164	78.1	45	21.4	1	0.5
	M2K	10	1.3	0	0.0	2	20.0	8	80.0	1	10.0	2	20.0	7	70.0	1	10.0	9	90.0	0	0.0
Potatoes, sweet potatoes and yams (e.g., French fries)	All	140	0.9	86	61.4	48	34.3	6	4.3	5	3.6	22	15.7	113	80.7	26	18.6	92	65.7	22	15.7
	M2K	4	0.5	4	100.0	0	0.0	0	0.0	0	0.0	0	0.0	4	100.0	0	0.0	3	75.0	1	25.0
Salads (e.g., Greek or macaroni)	All	70	0.5	10	14.3	32	45.7	28	40.0	1	1.4	3	4.3	66	94.3	3	4.3	47	67.1	20	28.6
	M2K	0	0.0																		
Sauces, dips, gravies and condiments (e.g., ketchup, hummus)	All	1224	8.1	374	30.6	397	32.4	453	37.0	83	6.8	45	3.7	1096	89.5	545	44.5	528	43.1	151	12.3
	M2K	0	0.0																		
Snacks (e.g., popcorn, chips)	All	813	5.3	347	42.7	299	36.8	167	20.5	133	16.4	123	15.1	557	68.5	74	9.1	448	55.1	291	35.8
	M2K	41	5.5	10	24.4	6	14.6	25	61.0	7	17.1	17	41.5	17	41.5	5	12.2	26	63.4	10	24.4
Soups (i.e., all varieties)	All	454	3.0	104	22.9	288	63.4	62	13.7	16	3.5	6	1.3	432	95.2	81	17.8	268	59.0	105	23.1
	M2K	1	0.1	0	0.0	1	100.0	0	0.0	0	0.0	0	0.0	1	100.0	0	0.0	1	100.0	0	0.0
Sugars and sweets (e.g., confectionary, chocolate, syrup)	All	734	4.8	20	2.7	4	0.5	710	96.7	431	58.7	284	38.7	19	2.6	381	51.9	88	12.0	265	36.1
	M2K	84	11.2	1	1.2	0	0.0	83	98.8	47	56.0	36	42.9	1	1.2	52	61.9	16	19.0	16	19.0
Vegetables (e.g., canned or frozen)	All	833	5.5	645	77.4	82	9.8	106	12.7	279	33.5	58	7.0	496	59.5	745	89.4	81	9.7	7	0.8
	M2K	0	0.0																		

^a^ Data were missing for sugars for n = 8 products, for sodium for n = 5 products and for saturated fat for n = 10 products, which were excluded from this analysis; ^b^ Foods in FLIP 2013 were classified into food categories as defined in the Table of Reference Amounts for Foods (TRA) in the Food and Drug Regulations [19]; ^c^ All = all products in FLIP (n = 15,200); M2K = products displaying child-appealing marketing on the packaging (n = 747); ^d^ Products that contained no “sugars”, “added sodium” or “added fat” were exempted from all “low in” thresholds; ^e^ Percentage of total products analyzed in the analytic sample (i.e., out of n = 15200 for “All” and out of n = 747 for “M2K”), note: some rows may sum to 100% plus or minus 0.1%, due to rounding; ^f^ Percentage of total products analyzed in that food category, in that analytic sample.
